# Mitochondrial Dysfunction Associates With Acute T Lymphocytopenia and Impaired Functionality in COVID-19 Patients

**DOI:** 10.3389/fimmu.2021.799896

**Published:** 2022-01-14

**Authors:** Yufei Mo, Kelvin Kai-Wang To, Runhong Zhou, Li Liu, Tianyu Cao, Haode Huang, Zhenglong Du, Chun Yu Hubert Lim, Lok-Yan Yim, Tsz-Yat Luk, Jacky Man-Chun Chan, Thomas Shiu-Hong Chik, Daphne Pui-Ling Lau, Owen Tak-Yin Tsang, Anthony Raymond Tam, Ivan Fan-Ngai Hung, Kwok-Yung Yuen, Zhiwei Chen

**Affiliations:** ^1^ AIDS Institute and Department of Microbiology, Li Ka Shing Faculty of Medicine, The University of Hong Kong, Pokfulam, Hong Kong SAR, China; ^2^ State Key Laboratory of Emerging Infectious Diseases, The University of Hong Kong, Pokfulam, Hong Kong SAR, China; ^3^ Center for Virology, Vaccinology and Therapeutics, Health@InnoHK, The University of Hong Kong, Hong Kong, Hong Kong SAR, China; ^4^ Department of Clinical Microbiology and Infection Control, The University of Hong Kong-Shenzhen Hospital, Shenzhen, China; ^5^ Department of Medicine and Geriatrics, Princess Margaret Hospital, Hong Kong, Hong Kong SAR, China; ^6^ Department of Medicine, The University of Hong Kong, Pokfulam, Hong Kong SAR, China

**Keywords:** mitochondrial dysfunction (MD), T-cell functionality, memory T cell, SARS-CoV-2, COVID-19

## Abstract

Severe acute respiratory syndrome coronavirus 2 (SARS-CoV-2) infection results in rapid T lymphocytopenia and functional impairment of T cells. The underlying mechanism, however, remains incompletely understood. In this study, we focused on characterizing the phenotype and kinetics of T-cell subsets with mitochondrial dysfunction (MD) by multicolor flow cytometry and investigating the association between MD and T-cell functionality. While 73.9% of study subjects displayed clinical lymphocytopenia upon hospital admission, a significant reduction of CD4 or CD8 T-cell frequency was found in all asymptomatic, symptomatic, and convalescent cases. CD4 and CD8 T cells with increased MD were found in both asymptomatic and symptomatic patients within the first week of symptom onset. Lower proportion of memory CD8 T cell with MD was found in severe patients than in mild ones at the stage of disease progression. Critically, the frequency of T cells with MD in symptomatic patients was preferentially associated with CD4 T-cell loss and CD8 T-cell hyperactivation, respectively. Patients bearing effector memory CD4 and CD8 T cells with the phenotype of high MD exhibited poorer T-cell responses upon either phorbol 12-myristate-13-acetate (PMA)/ionomycin or SARS-CoV-2 peptide stimulation than those with low MD. Our findings demonstrated an MD-associated mechanism underlying SARS-CoV-2-induced T lymphocytopenia and functional impairment during the acute phase of infection.

## Introduction

Severe acute respiratory syndrome coronavirus 2 (SARS-CoV-2), the causative agent of the coronavirus disease 2019 (COVID-19) (1), has shocked the world with more than 240 million confirmed cases and more than 4.9 million deaths by October 20, 2021, as reported by the World Health Organization (2). SARS-CoV-2 became the most devastating human coronavirus, which was associated with its extremely high transmissibility (3, 4), hidden asymptomatic spread (4, 5), and emerged viral variants of concern escaping from neutralizing antibodies during natural infection or post vaccination (6–8). Clinically, COVID-19 patients might develop acute respiratory distress syndrome or be admitted to the intensive care unit (ICU) at around 8–15 days post symptom onset (p.s.o.) (9, 10). Most severe COVID-19 patients also suffered from prompt lymphocytopenia (11). Low T-cell count on hospital admission may predict disease severity (12) and relate to the increased peripheral pro-inflammatory cytokines among COVID-19 patients (13, 14). Particularly, the low CD8 T-cell count has been suggested to be a predictor for high mortality and severity of COVID-19 pneumonia (13, 15, 16). Furthermore, T-cell lymphocytopenia is associated with higher saliva viral load (17). These findings demonstrated that T lymphocytopenia might have detrimental effects on acute COVID-19 patients. Considering that T-cell immunity is important for host immune defense by eliminating virus-infected cells and assisting antibody responses (18, 19), we sought to investigate possible causes of T lymphocytopenia during the natural course of acute SARS-CoV-2 infection. We focused on the characterization of T-cell subsets with mitochondrial dysfunction (MD) by flow cytometry and the investigation of the association between MD and T-cell functionality.

## Materials and Methods

### Study Subjects

Our study was approved by the institutional review board of the University of Hong Kong/Hospital Authority Hong Kong West Cluster and Kowloon West Cluster Research Ethics Committee [UW 13-265 and KW/EX-20-038(144-26)]. We recruited 88 acute patients (APs), admitted to the Queen Mary Hospital and Princess Margaret Hospital from August 6, 2020, to December 23, 2020, into our study. In the meantime, 17 convalescent patients (CPs) and 31 healthy donors (HDs) were recruited by the Hong Kong Red Cross as controls. We primarily divided APs into three groups: asymptomatic (AS), mild APs (symptomatic without O_2_ treatment), and severe APs (symptomatic with O_2_ treatment). The clinical information of our subjects was summarized in **Table 1**.

**Table 1:** Brief table for patient information description of 88 recruited acute patients.

Characteristics	Severe (n = 26)	Non-severe (n = 62)	*p* value
**Demographic**			
Age, median years (interquartile range)	62 (52–70)	60 (42–67)	0.224
Female	7 (26.9)	35 (56.5)	0.018
**Chronic comorbidities**			
Hypertension	7 (26.9)	15 (24.2)	0.792
Chronic heart disease	3 (11.5)	5 (8.1)	0.689
Chronic lung disease	2 (7.7)	2 (3.2)	0.578
Chronic kidney disease	1 (3.8)	2 (3.2)	1.000
Diabetes mellitus	6 (23.1)	9 (14.5)	0.361
Other endocrine or metabolic diseases	3 (11.5)	3 (4.8)	0.355
Neurological disease	4 (15.4)	4 (6.5)	0.228
Any chronic comorbidities	16 (61.5)	32 (51.6)	0.484
**Blood tests on admission** (median, interquartile range)			
Hemoglobin (g/dl)	14.2 (13.6–15.1)	13.8 (13.0–14.6)	0.09
Total white blood cell count (×10^9^/L)	5.9 (5.0–7.2)	4.8 (4.0–5.9)	0.007
Neutrophil count (×10^9^/L)	4.1 (3.2–5.2)	3.0 (2.2–4.0)	0.001
Lymphocyte count (×10^9^/L)	1.05 (0.75–1.27)	1.11 (0.83–1.62)	0.186
Platelet count (×10^9^/L)	189 (133–235)	201 (163–255)	0.202
**Initial viral load**			
Ct value	21.4 (16.4–25.6)	23.1 (17.8–28.9)	0.242
**Severity**			
Oxygen supplementation	26 (100)	0 (0)	<0.001
ICU admission	10 (38.5)	0 (0)	<0.001
Death	1 (3.8)	0 (0)	0.295

ICU, intensive care unit.

### Peripheral Blood Mononuclear Cell Isolation

Fresh peripheral blood mononuclear cells (PBMCs) from HDs and patients were isolated using Lymphoprep™ (Axis Shield, Dundee, Scotland) density gradient centrifugation in our BSL-3 laboratory. Freshly purified PBMCs were used for phenotyping by flow cytometry, T-cell proliferation assay, T-cell functionality assay, and/or antigen-specific assay. For all experiments, PBMCs were cultured in R10 Medium [RPMI 1640 medium (Gibco™, Thermo Fisher, MA, USA) supplemented with 10% fetal bovine serum (FBS; Gibco™), 2 mM L-glutamine (Gibco™), and 100 U/ml penicillin/100 μg/ml streptomycin (Gibco™)] with 5% CO_2_ at 37°C. The rest of the cells were kept in freezing medium [90% FBS + 10% dimethylsulfoxide (DMSO; Sigma Aldrich, MA, USA)] at -150°C before use. Frozen PBMCs were recovered in pre-warmed R10 Medium with 5% CO_2_ at 37°C overnight.

### Flow Cytometry

Cells from fresh or frozen samples were stained with mitochondrial indicators including MitoTracker™ Green FM (200 nM, 37°C for 15 min), MitoTracker™ Red CMXRos (200 nM, 37°C for 15 min), tetramethylrhodamine methyl ester **(**TMRM; 125 nM, 37°C for 15 min), and MitoSOX™ (5 µM, 37°C for 10 min) before surface antibody staining (**Table 2**). After cells were washed twice using the staining buffer [2% FBS in PBS (Gibco™)], they were stained with Zombie Aqua™ Kit, FACS antibodies, and calcium indicator Fluo-4FF (**Table 2**) in the staining buffer accordingly at 4°C for 30 min. For intracellular staining, cells were fixed and permeabilized using Fixation/Permeabilization Solution (BD Sciences, NJ, USA) at 4°C for 20 min, followed by washing with Perm/Wash™ buffer (BD Sciences) and then stained with cytokine antibodies (**Table 2**) at 4°C overnight. Cells were washed twice before FACS analysis. For the Annexin V assay, T cells were stained with PE/Cyanine7 Annexin V (**Table 2**) in the Annexin V binding buffer (BD Sciences) before FACS analysis.

**Table 2:** List of antibodies or reagents that were used for FACS analysis.

Reagent	Source	Identifier
MitoTracker™ Green FM	Thermo Fisher	Cat. No: M7514
MitoTracker™ Red CMXRos	Thermo Fisher	Cat. No: M7512
TMRM assay kit	Abcam	Cat. No: ab228569
MitoSOX™ Red Mitochondrial Superoxide Indicator	Thermo Fisher	Cat. No: M36008
Fluo-4FF, AM, cell permeant	Thermo Fisher	Cat. No: F23981
Zombie Aqua™ Kit	BioLegend	Cat. No: 423102
PE/Cyanine7 Annexin V	BioLegend	Cat. No: 640950
Brilliant Violet 711™ anti-human CD3 Antibody	BioLegend	Cat. No: 317328; RRID : AB_2562907
Brilliant Violet 785™ anti-human CD3 Antibody	BioLegend	Cat. No: 344842; RRID : AB_2616891
PerCP/Cyanine5.5 anti-human CD4 Antibody	BioLegend	Cat. No: 317428; RRID : AB_1186122
PE/Dazzle™ 594 anti-human CD8a Antibody	BioLegend	Cat. No: 301058; RRID : AB_2563570
APC anti-human CD38 Antibody	BioLegend	Cat. No: 356606; RRID : AB_2561902
Brilliant Violet 421™ anti-human CD45RA Antibody	BioLegend	Cat. No: 304130; RRID : AB_10965547
APC/Cyanine7 anti-human CD197 (CCR7) Antibody	BioLegend	Cat. No: 353212; RRID : AB_10916390
PE/Cyanine7 anti-human CD8 Antibody	BioLegend	Cat. No: 344712; RRID : AB_2044008
APC/Fire™ 750 anti-human CD8 Antibody	BioLegend	Cat. No: 344746; RRID: AB_2572095
Brilliant Violet 605™ anti-human CD279 (PD-1) Antibody	BioLegend	Cat. No: 329924; RRID : AB_2563212
PE/Cyanine7 anti-human IL-2 Antibody	BioLegend	Cat. No: 500326; RRID : AB_2125593
FITC anti-human TNF-α Antibody	BioLegend	Cat. No: 502906; RRID : AB_315258
PE anti-human IFN-γ Antibody	BioLegend	Cat. No: 506507; RRID : AB_315440
Brilliant Violet 785™ anti-human HLA-DR Antibody	BioLegend	Cat. No: 307642; RRID : AB_2563461

### T-Cell Proliferation Assay

Fresh PBMCs were pre-labeled with carboxyfluorescein 6 succinimidyl ester (CFSE; 5 µM; Thermo Fisher) at 37°C for 10 min. Cells were then stimulated with anti-CD3 (2 µg/ml; BioLegend, CA, USA) plus anti-CD28 (1 µg/ml; BioLegend) antibodies for 3 days before FACS analysis. Non-stimulated or non-labeled cells served as controls. Proliferation index was calculated by normalizing the total number of divisions to number of cells that went into division (referring to flowjo proliferation platform: https://docs.flowjo.com/flowjo/experiment-based-platforms/proliferation/).

### Polyfunctional Assay in T Cells

Fresh PBMCs from patients or HDs were stimulated with the commercially available 500× cell activation cocktail [BioLegend; containing phorbol 12-myristate-13-acetate (PMA, 40.5 µM) and ionomycin (669.3 µM)] in the presence of brefeldin A (BFA; 7.5 µg/ml; Sigma-Aldrich) in R10 Medium for 6 h. Cells were then harvested for intracellular FACS analysis on tumor necrosis factor (TNF)α, interferon (IFN)γ, and interleukin (IL)-2 expression.

### Antigen-Specific Assay in T Cells

As previously described (20, 21), fresh PBMCs were stimulated by 1 µg/ml spike peptide pool or 5 µg/ml nucleocapsid protein (NP) peptide pool of SARS-CoV-2 (15-mer overlapping by 11, spanning the whole spike or NP; Genscript, NJ, USA) in the presence of 0.5 µg/ml anti-CD28 and anti-CD49d antibodies (BioLegend) overnight. PMA/ionomycin stimulation served as positive control, while media only served as negative control. BFA (7.5 µg/ml) was added at 6 h before harvesting cells for intracellular FACS analysis on IFNγ expression.

### Immunofluorescence Confocal Microscopy

Valinomycin-treated PBMCs from HDs (1 µM, 60 min) and thawed PBMCs from HDs or APs were stained with MitoTracker™ Green (200 nM) and MitoTracker™ Red CMXRos (200 nM) at 37°C for 15 min before blocking with the Fc Blocker (BioLegend). Then, cells were washed and stained with anti-CD3 antibody (DAKO, 1:50) at 4°C for 30 min followed by secondary AF647-conjugated antibody staining at 4°C for 30 min. After being washed and stained with Hoechst33342 buffer (Thermo Fisher), cells were transferred onto Nunc™ Lab-Tek™ II 8-well chambered coverglass (Thermo Fisher) for confocal analysis.

### Seahorse XF Cell Mito Stress Test

Around 0.5 million purified total T cells from fresh PBMCs or thawed PBMCs of HDs or APs were treated with or without 1 µM valinomycin for 60 min before performing Seahorse XF Cell Mito Stress Test. Experimental procedure was strictly based on the Seahorse XF Cell Mito Stress Test Kit [Oligomycin 15 µM, Carbonyl cyanide 4-(trifluoromethoxy)phenylhydrazone (FCCP) 15 µM, Antimycin A/Rotenone stock 5 µM; Agilent]. Seahorse XFe96 Analyzer was used for detecting oxygen consumption rate (OCR).

### Statistical Analysis

All statistical analyses were performed using the SPSS or GraphPad Prism 7 software. Data represent mean or mean with SEM of at least three independent experiments unless indicated. To compare the frequency of MD^+^ cells between patients with high and low levels of markers (including HLA-DR^+^CD38^+^, PD-1^+^, and TNFα^+/-^IFN**γ** ^+/-^IL-2^+/-^) or proliferation index, patients with level higher than the median were regarded as the high group, while others as the low group. Significant differences were calculated using a one-way analysis of variance (ANOVA) or two-tailed unpaired Student’s *t* test. *p* < 0.05 was considered statistically significant.

## Results

### Acute SARS-CoV-2 Infection Results in Rapid Mitochondrial Dysfunction in Both CD4 and CD8 T Cells

By assessing clinical test results, we found that 73.9% of the 88 recruited study subjects displayed lymphocytopenia upon hospital admission. Consistent with previous findings, men were more likely to become severely ill than women (**Table 1**) (22, 23). A higher neutrophil count was found in severe APs than non-severe ones (**Table 1**) (24). There were no differences between recruited mild and severe APs in terms of other clinical presentations (**Table 1**).

**Figure 1:**
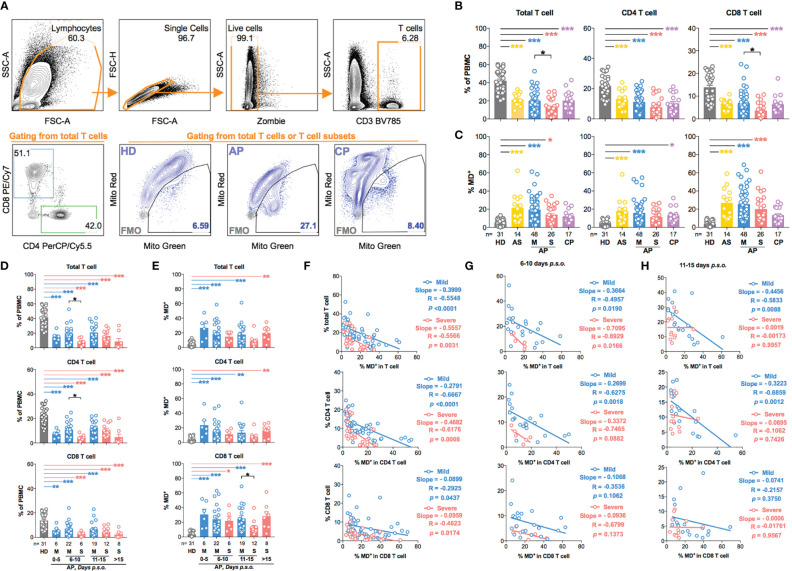
T-cell subsets in asymptomatic and symptomatic donors are characterized by increased production of dysfunctional mitochondria during SARS-CoV-2 acute infection. Fresh peripheral blood mononuclear cells (PBMCs) of 88 SARS-CoV-2 acute patients (APs), 17 convalescent patients (CPs), and 31 healthy donors (HDs) were collected and profiled using flow cytometry in at least three independent experiments. **(A)** Representative plots showed the gating strategy on different T-cell subsets and cells with mitochondrial dysfunction (MD^+^) based on fluorescence minus one (FMO) control in FACS analysis. **(B)** The frequency of total T cells, CD4 T cells, and CD8 T cells and **(C)** the percentage of MD^+^ cells in total T cells, CD4 T cells, and CD8 T cells were compared among different groups based on disease severity compared to HDs and CPs. For symptomatic patients, **(D)** the frequency of total T cells, CD4 T cells, and CD8 T cells and **(E)** the percentage of MD^+^ cells in total T cells, CD4 T cells, and CD8 T cells along disease development were compared between mild and severe APs. HDs (in gray) served as negative control. Data represent mean ± SEM. Statistics were calculated based on one-way ANOVA test. *p < 0.05, **p < 0.01, ***p < 0.001. **(F)** The correlation between cell frequency and the percentage of MD^+^ cells in T-cell subsets from mild or severe APs during all time periods was calculated by Pearson’s correlation coefficient analysis. The correlation between cell frequency and the percentage of MD^+^ cells in T-cell subsets from mild or severe APs during the period of 6–10 days **(G)** or 11–15 days **(H)** post symptom onset (p.s.o.) was also calculated by Pearson correlation coefficient analysis.

We then profiled T-cell frequency in APs [n = 88; 14 asymptomatic (AS), 48 mild APs, and 26 severe APs], as compared to the control groups including HDs (n = 31) and CPs (n = 17), by FACS analysis (**Figure 1A**). Consistent with results as previously described by us and others (13, 21), T-cell frequency in APs declined dramatically as compared with HDs (**Figure 1B**). More CD8 T-cell reductions were found among severe APs (**Figure 1B**) than mild ones (15). In the meantime, we used MitoTracker™ Green (Mito Green, representing for mitochondrial density) and MitoTracker™ Red CMXRos (Mito Red, representing for mitochondrial membrane potential) to assess mitochondrial function by flow cytometry. The MD^+^ proportion (**Figure 1A**) was defined by cells of Mito Green^high^ Mito Red^Low^ (25–27), which was validated by confocal study and Seahorse XF Cell Mito Stress Test using the MD-inducing drug, valinomycin, on HD PBMCs (**Supplementary Figures S1A**–**E**). As compared with HDs, T cells from both AS and symptomatic AP groups showed high frequencies of MD^+^ cells (**Figure 1C**). Moreover, the frequencies of MD^+^ CD8 T cells were relatively higher than those of MD^+^ CD4 T cells in both mild and severe APs (**Figure 1C**). The elevated MD^+^ T cell proportion was consolidated by correlation analysis with the Mito Green^high^ TMRM^Low^ population [TMRM, another indicator for mitochondrial membrane potential (28)]. Both Mito Green^high^ Mito Red^Low^ and Mito Green^high^ TMRM^Low^ correlated positively with the Annexin V^+^ apoptotic proportion in CD4 or CD8 T cells from APs (**Supplementary Figures S2A, B**). Besides, the phenotype of MD^+^ T cells was supported by moderately less fission under confocal analysis (29) and by relatively lower ATP production and maximal respiration by Seahorse XF Cell Mito Stress Test (**Supplementary Figures S3A**–**F**).

Subsequently, we analyzed the kinetics of MD^+^ T cells among symptomatic patients. In the first 5 days p.s.o., frequencies of T-cell subsets decreased quickly, while proportions of MD^+^ T-cell subsets increased (**Figures 1D, E**). MD^+^ T-cell proportion, therefore, was negatively correlated with T-cell frequency, especially with a steeper slope of curve in CD4 than CD8 T-cell subsets (**Figure 1F**). Significant reduction of CD4 T-cell frequency was found in severe APs during the period of 6–10 days p.s.o., albeit that a stronger negative correlation between MD^+^ proportion and CD4 T-cell frequency occurred in mild APs than severe APs (**Figures 1D, G**). During 11–15 days p.s.o., significantly lower MD^+^ proportion in CD8 T cells was found in severe than mild APs, although no significant correlation was found between MD^+^ proportion and CD8 T-cell frequency (**Figures 1E, H**). These results demonstrated that acute SARS-CoV-2 infection resulted in significant CD4 T-cell loss and subsequent CD8 T-cell loss especially among severe APs, and that MD correlated more negatively with CD4 than CD8 T-cell loss regardless of disease severity.

### COVID-19 Patients With Hyperactivated Memory T Cells Display High Mitochondrial Dysfunction Phenotype

**Figure 2:**
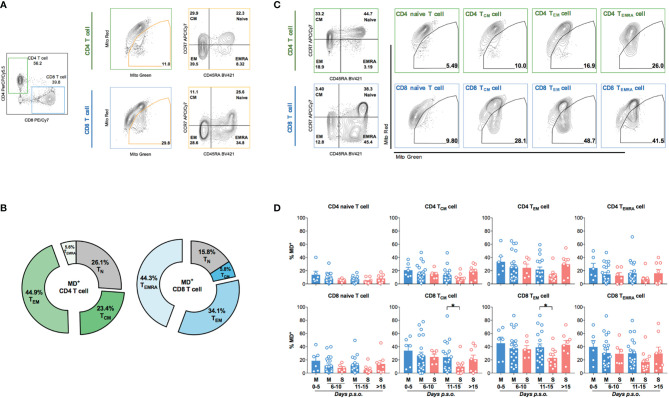
Higher percentage of MD^+^ cells is found in memory CD8 T cell from mild patients than severe patients at 11-15 days p.s.o. during SARS-CoV-2 infection. Eighty-eight SARS-CoV-2 acute patients (APs) were collected for data analysis on T-cell subsets among cells with mitochondrial dysfunction (MD^+^) using flow cytometry in at least three independent experiments. **(A)** Representative plots showed the gating strategy on Naive T (CD45RA^+^CCR7^+^), central memory T (T_CM_, CD45RA^-^CCR7^+^), effector memory T (T_EM_, CD45RA^-^CCR7^-^), and effector memory T-cell re-expressing CD45RA (T_EMRA_, CD45RA^+^CCR7^-^) from MD^+^ CD4 or CD8 T cells. **(B)** The average proportions of different T-cell subsets in MD^+^ CD4 T-cell and CD8 T-cell subsets were calculated and displayed (number represents mean) in pie charts. Seventy-three SARS-CoV-2 APs [47 mild (M) and 26 severe (S) ones; only one mild AP on Day 16 post symptoms onset (p.s.o.) was excluded for statistical analysis] were collected for data analysis on MD^+^ cells in various T-cell subsets using flow cytometry in at least three independent experiments. **(C)** Representative plots showed the gating strategy on MD^+^ cells from Naive T, T_CM_, T_EM_, and T_EMRA_ subsets from CD4 or CD8 T cells. **(D)** The percentages of MD^+^ cells in Naive T, T_CM_, T_EM_, and T_EMRA_ subsets from CD4 T cells and CD8 T cells along disease development were compared between mild and severe APs. Data represent mean ± SEM. Statistics were calculated based on unpaired Student’s t-test. *p < 0.05.

**Figure 3:**
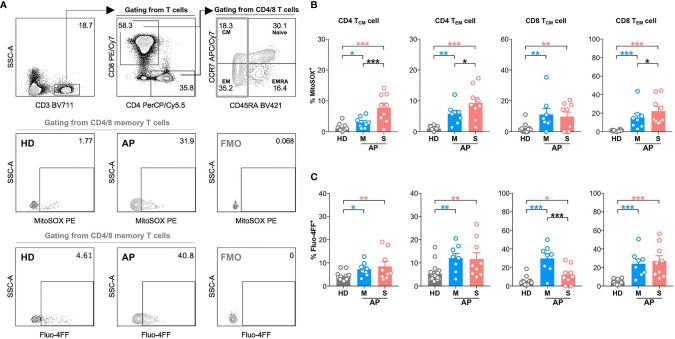
High mitochondrial reactive oxygen species (ROS) level and intracellular calcium level are found in memory CD4 or CD8 T cells at 11-15 days p.s.o. during SARS-CoV-2 infection. Seventeen SARS-CoV-2 acute patients [APs; 8 mild (M) and 9 severe (S) ones on Day 11–15 post symptom onset (p.s.o.)] and 18 healthy donors (HDs) were collected for FACS analysis in at least three independent experiments. **(A)** Representative plots showed the gating strategy on MitoSOX^+^ cells and Fluo-4FF^+^ cells among memory CD4 or CD8 T cells. The percentages of MitoSOX^+^ cells **(B)** and Fluo-4FF^+^ cells **(C)** in CD4 T_CM_, CD4 T_EM_, CD8 T_CM_, and CD8 T_EM_ cells from mild and severe APs on Day 11–15 p.s.o. were displayed in bar charts. HDs served as control. Data represent mean ± SEM. Statistics were calculated based on one-way ANOVA test. *p < 0.05, **p < 0.01, ***p < 0.001.

Next, we further analyzed MD phenotype in various T-cell subsets. On average, we found that both central (23.4%) and effector (44.9%) memory CD4 T cells (CD4 T_CM_ and CD4 T_EM_) were among total MD^+^ CD4 T cells (**Figures 2A, B**). Similarly, both effector memory (34.1%) and effector (44.3%) CD8 T cells were among total MD^+^ CD8 T cells (**Figures 2A, B**). At around 11–15 days p.s.o., albeit with higher percentage of MitoSOX^+^ [representing mitochondrial reactive oxygen species (ROS)] cells, significantly lower MD^+^ central and effector memory CD8 T cells (CD8 T_CM_ and CD8 T_EM_) were shown in severe APs than mild APs (**Figures 2C, D**; **3A, B**). Similar changes of MD level were not found in CD4 T_CM_ and CD4 T_EM_ cells despite higher mitochondrial ROS level in severe APs than mild APs (**Figures 2C, D**; **3A, B**). Along with MD and MitoSOX phenotype, Fluo-4FF^+^ [representing intracellular calcium level] cells were also maintained at a high level in these four T-cell subsets from both mild and severe APs (**Figures 3A, C**).

**Figure 4:**
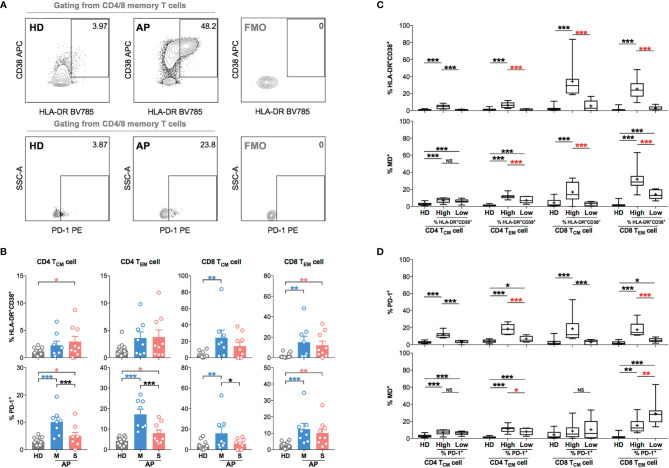
Memory CD4 or CD8 T cells with increased level of T-cell hyperactivation exhibit higher MD^+^ proportion at 11-15 days p.s.o. during SARS-CoV-2 infection. In order to understand the relationship between cells with mitochondrial dysfunction (MD^+^) and T-cell activation in COVID-19 patients, 17 SARS-CoV-2 acute patients [APs; 8 mild (M) and 9 severe (S) ones on Day 11–15 p.s.o.] and 18 healthy donors (HDs) were collected for FACS analysis in at least three independent experiments. **(A)** Representative plots showed the gating strategy on HLA-DR^+^CD38^+^ and PD-1^+^ cells among central memory (CM) or effector memory (EM) subsets in CD4 or CD8 T cells. **(B)** The percentages of HLA-DR^+^CD38^+^ and PD-1^+^ cells in CD4 T_CM_, CD4 T_EM_, CD8 T_CM_, and CD8 T_EM_ cells from mild and severe APs on Day 11–15 p.s.o. were displayed in bar charts. HDs served as control. **(C)** To investigate the relationship between MD^+^ proportion and T-cell activation, the percentages of HLA-DR^+^CD38^+^ cells and MD^+^ proportion in CD4 T_CM_, CD4 T_EM_, CD8 T_CM_, and CD8 T_EM_ cells were compared between patients with a high percentage of HLA-DR^+^CD38^+^ cells (higher than median) and those with a low percentage of HLA-DR^+^CD38^+^ cells (not higher than median). **(D)** Similarly, the percentages of PD-1^+^ cells and MD^+^ proportion in CD4 T_CM_, CD4 T_EM_, CD8 T_CM_, and CD8 T_EM_ cells were compared between patients with high percentage of PD-1^+^ cells (higher than median) and those with low percentage of PD-1^+^ cells (not higher than median). HDs served as control. Data represent mean ± SEM. Statistics were calculated based on one-way ANOVA test. *p < 0.05, **p < 0.01, ***p < 0.001. NS, not significantly different.

To further investigate the role of MD in regulating T-cell activation and function, we examined biomarkers of T-cell hyperactivation (HLA-DR^+^CD38^+^) and activation/exhaustion (PD-1^+^) in memory subsets along with monitoring MD in APs (8 mild and 9 severe APs during 11–15 days p.s.o.) as compared to HD controls (**Figure 4A**). Like previous findings (21), CD4 T_CM_, CD4 T_EM_, CD8 T_CM_, and CD8 T_EM_ cells from APs showed moderately increased percentage of HLA-DR^+^CD38^+^ cells and significantly increased percentage of PD-1^+^ cells as compared to HD (**Figure 4B**). Mild APs were likely to have higher frequencies of PD-1^+^ CD4 T_CM_, CD4 T_EM_, and CD8 T_CM_ cells than severe ones (**Figure 4B**). Interestingly, when comparing MD^+^ vs. HLA-DR^+^CD38^+^ cells, APs with highly hyperactivated CD4 T_EM_, CD8 T_CM_, and CD8 T_EM_ cells also exhibited significantly higher MD^+^ (**Figure 4C**). For PD-1 expression, however, PD-1^+^ memory T cells showed little association with MD^+^ proportion except for T_EM_ cells (**Figure 4D**). These results indicated that APs with hyperactivated memory T cells, especially memory CD8 T cells, displayed high MD phenotype at the time of 11–15 days p.s.o.. These findings implicated that mitochondria were likely involved in regulating T-cell hyperactivation and immune responses at the later stage of SARS-CoV-2 infection.

### High Mitochondrial Dysfunction Is Associated With Reduced T_EM_ Cell Functionality in COVID-19 Patients

**Figure 5:**
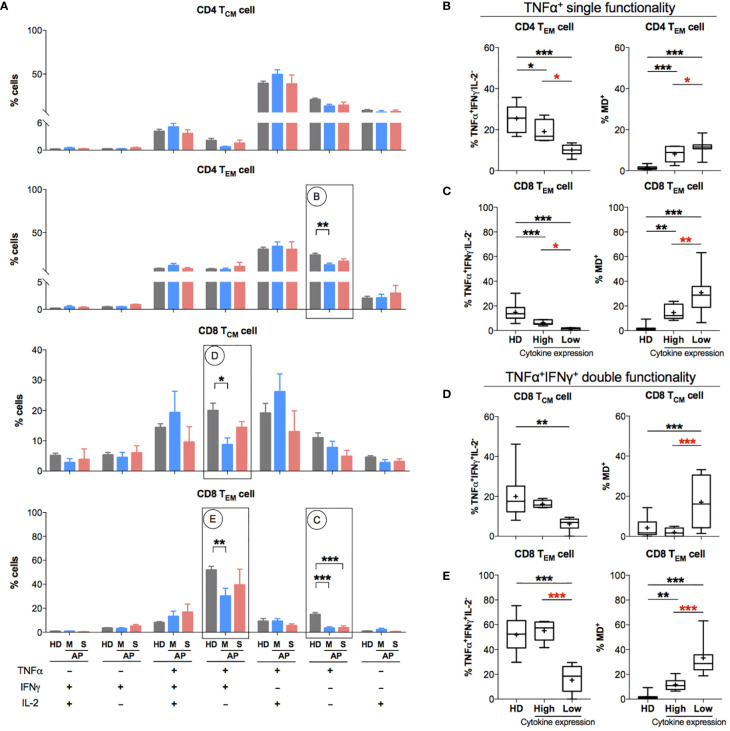
Patients with impaired functionality in effector memory T cells have high MD^+^ proportion. In order to understand the relationship between cells with mitochondrial dysfunction (MD^+^) and phorbol 12-myristate-13-acetate (PMA)-stimulated T-cell responses in COVID-19 patients at the later stage of infection, 13 SARS-CoV-2 acute patients [APs; 8 mild (M) and 5 severe (S) on Day 11–15 post symptom onset (p.s.o.)] and 18 healthy donors (HDs) were collected for polyfunctional analysis in at least three independent experiments. Fresh peripheral blood mononuclear cells (PBMCs) from these 13 recruited APs were treated with PMA/ionomycin in the presence of brefeldin A (BFA) for 6 h. PBMCs from 18 HDs served as control. Cells were then harvested for FACS analysis on intracellular expression of TNFα, IFNγ, and IL-2. BFA-treated only cells served as negative control. **(A)** The percentages of TNFα^+/-^IFNγ^+/-^IL2^+/-^ cells in CD4 T_CM_, CD4 T_EM_, CD8 T_CM_, and CD8 T_EM_ cells were displayed in bar charts. The groups of TNFα^+/-^IFNγ^+/-^IL2^+/-^ cells [labeled with **(B–E)** in cycle] with significant changes in APs were selected for further comparison as shown in the corresponding figures **(B–E)**. The percentage of certain cytokine expression groups and profile of MD^+^ proportion in CD4 T_EM_, CD8 T_CM_, and CD8 T_EM_ cells were compared between patients with highly cytokine-expressed cells (higher than median) and those with impaired cytokine-expressed cells (not higher than median). Data represent mean ± SEM. Statistics were calculated based on one-way ANOVA test. *p < 0.05, **p < 0.01, ***p < 0.001.

Since memory T-cell hyperactivation was likely to be associated with high MD in APs at the time of 11–15 days p.s.o., we investigated the relationship between MD and T-cell functionality at this stage. After 3-day non-antigen T-cell receptor (TCR) activation, PBMCs from APs (8 mild and 5 severe) and HDs were harvested for proliferation analysis. Significantly lower proliferation index was found in both CD4 and CD8 T cells of mild rather than severe APs (**Supplementary Figures S4A, B**). Moreover, APs with lower proliferation index in CD8 T cells showed significantly higher MD, which was not shown in CD4 T cells (**Supplementary Figures S4C, D**). Subsequently, we analyzed T-cell functionality upon 6-h non-antigen PMA/ionomycin activation on PBMCs from these APs by evaluating intracellular cytokine expression levels of TNFα, IFNγ, and IL-2 (21, 30) (**Supplementary Figure 5**). Both CD4 T_EM_ and CD8 T_EM_ cells from mild APs exhibit a significantly lower percentage of single functional TNFα^+^IFNγ^-^IL2^-^ cells compared to HDs (**Figure 5A**). Similarly, lower percentage of dual functional TNFα^+^IFNγ^+^IL2^-^ cells was detected in both CD8 T_CM_ and CD8 T_EM_ cells from mild APs, which was not found in severe APs (**Figure 5A**). Among aforementioned functionally defective subsets of memory T cells, dramatically higher MD^+^ proportion was observed in those subsets with lower percentage of single functional TNFα^+^IFNγ^-^IL2^-^ cells (**Figures 5B, C**) or dual functional TNFα^+^IFNγ^+^IL2^-^ cells (**Figures 5D, E**). Basically, MD in T_EM_ cells of APs was associated with reduced T-cell functionality upon PMA/ionomycin activation.

### Patients With Poor Antigen-Specific CD4 T_EM_ Cell Response Exhibit High MD^+^ CD4 T_EM_ Cell

**Figure 6:**
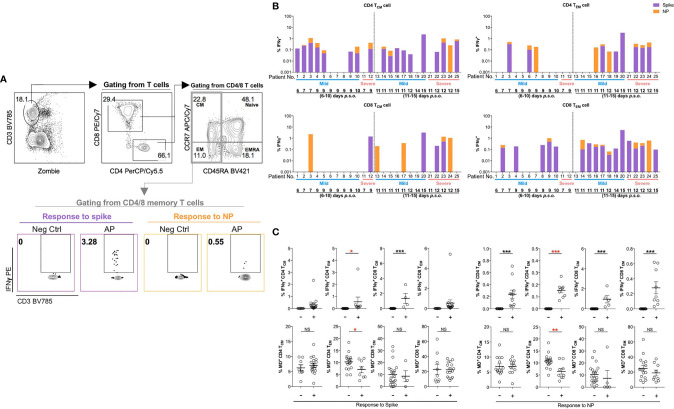
Patients with poor SARS-CoV-2-specific responses in effector memory CD4 T cells show significantly higher MD^+^ proportion. In order to understand the relationship between cells with mitochondrial dysfunction (MD^+^) and SARS-CoV-2-specific T-cell responses in COVID-19 patients, 25 SARS-CoV-2 acute patients [APs; 18 mild (M) and 7 severe (S) ones during time periods of 6–10 and 11–15 days post symptoms onset (p.s.o.)] were collected for antigen-specific response assessment in at least three independent experiments. Fresh peripheral blood mononuclear cells (PBMCs) were treated with 1 µg/ml spike peptide pool or 5 µg/ml purified nucleocapsid protein (NP) peptide pool in the presence of 0.5 µg/ml anti-CD28 and anti-CD49d antibodies overnight. Brefeldin A (BFA) was added 6 h before cells were harvested for FACS analysis on intracellular IFNγ level. **(A)** Representative plots displayed gating strategy on IFNγ^+^ cells among central memory (CM) or effector memory (EM) subsets in CD4 or CD8 T cells in response to spike or NP. **(B)** For these 25 patients, the percentages of IFNγ^+^ cells in CD4 T_CM_, CD4 T_EM_, CD8 T_CM_, or CD8 T_EM_ cells in response to spike (in purple) or NP (in orange) were displayed in stacked bar chart. Each column represents one individual. Data represent the exact value. **(C)** The percentages of IFNγ^+^ cells and MD^+^ cells in CD4 T_CM_, CD4 T_EM_, CD8 T_CM_, or CD8 T_EM_ cells from these 25 patients during the time period of 6–15 days p.s.o. were also compared between poor responders (-) and good responders (+) specific to spike or NP peptide pool. Data represent mean ± SEM. Statistics were calculated based on unpaired Student’s t-test. *p < 0.05, **p < 0.01, ***p < 0.001. NS, not significantly different.

Considering that acute SARS-CoV-2 infection preferentially induced antigen-specific CD4 T-cell response (21, 31), we investigated such responses in memory T-cell subsets upon restimulation using the peptide pool of spike or NP of SARS-CoV-2. Fresh PBMCs from APs (18 mild and 7 severe APs at 6–10 and 11–15 days p.s.o.) and HDs were stimulated by peptide pool of SARS-CoV-2 spike and NP, respectively, overnight and then were analyzed for IFNγ expression by flow cytometry (**Figure 6A**). Memory T cells from majority of APs at 11–15 days p.s.o. showed responses to spike and NP (**Figure 6B** and **Supplementary Figure S6A**), with low viral load (CT value ≥25) (**Supplementary Figure S6B**) (32). We also compared the MD^+^ proportion in these subsets specific to spike or NP among these 25 APs. Surprisingly, poor responders to antigen-specific responses of CD4 T_EM_ exhibited significantly higher MD^+^ proportion, which was not shown in CD4 T_CM_, CD8 T_CM_, and CD8 T_EM_ cells (**Figure 6C**). To avoid bias on days p.s.o., we separated the APs collected on Day 6–10 and 11–15 p.s.o. for comparison. Similarly, in APs on Day 11–15 p.s.o., patients bearing CD4 T_EM_ cells with defective NP-specific responses exhibited profoundly high MD^+^ proportion (**Supplementary Figure S7**). Therefore, MD in CD4 T_EM_ cells in APs was preferentially related to poor SARS-CoV-2-specific responses.

## Discussion

In this study, we demonstrated that SARS-CoV-2 infection resulted in rapidly increased frequencies of MD^+^ T cells by testing freshly isolated PBMCs from COVID-19 patients. Notably, this increase took place among all infected individuals including asymptomatic subjects within the first week of symptom onset and the symptomatic subjects without any drug treatment. Moreover, this increase was not restricted to a particular T-cell population because both CD4 and CD8 T cells with various phenotypic subsets displayed elevated MD. Among symptomatic patients, however, the frequency of MD^+^ T cells was preferentially correlated with CD4 T-cell loss and CD8 T-cell hyperactivation, respectively. While lower proportion of MD^+^ memory CD8 T cells was found among severe patients than in mild ones at the stage of disease severity, both mild and severe patients bearing effector memory MD^hi^ CD4 or MD^hi^ CD8 T cells exhibited poorer T-cell responses upon stimulation of either SARS-CoV-2 peptide or PMA/ionomycin. Our results, therefore, demonstrated that MD was associated with T lymphocytopenia and impaired T-cell functionality during acute phase of COVID-19.

SARS-CoV-2 manipulated host T-cell mitochondria rapidly, contributing to T lymphocytopenia. The rapid burst of viral loads during the first week of symptom onset might have direct effects on mitochondria. SARS-CoV-2 proteins nsp2 and nsp4 were likely involved in endoplasmic reticulum calcium homeostasis and mitochondrial biogenesis (33). SARS-CoV-2 RNA–protein interactions mediate virus-mediated mitochondrial dysfunction during early infection (34). Growing evidence indicated that the causes of this rapid manipulation might involve multiple mechanisms in immune cells. Recently, elevated mitochondrial mass was found to be correlated with mitochondrial apoptosis in T cells from COVID-19 patients (35). A highly expressed voltage-dependent anion channel 1, a mitochondrial membrane protein for transporting calcium, was suggested as a cause of mitochondrial dysregulation and apoptosis of T cells in COVID-19 patients (36). Moreover, COVID-19 patients displayed depolarized mitochondria and abnormal mitochondrial ultrastructure in monocytes, which was associated with the production of several inflammatory cytokines and chemokines (37). Inflammatory cytokines such as high levels of TNFα and IL-6 might then drive mitochondria-mediated T-cell apoptosis and lymphocytopenia (14, 38). Consistently with other studies (35, 36, 38), we observed a strongly negative correlation between profound T-cell loss and rapidly increased MD^+^ T-cell proportion. Besides, with a large number of patients, our results further extend the impact of SARS-CoV-2 infection on the kinetics of T-cell frequency and mitochondrial dysfunction not only to asymptomatic subjects but also to a much broader range of T-cell subsets. It is, therefore, plausible that mitochondria-driven T-cell apoptosis could be one of the mechanisms underlying clinical T lymphocytopenia.

SARS-CoV-2-induced mitochondrial dysfunction compromised T-cell functionality, contributing to suppressed T-cell immune responses to viral infection. Specific T-cell immune responses are essential for eliminating SARS-CoV-2-infected cells and assisting antibody responses (18, 19). Upon antigen binding to TCR, T cells undergo drastic metabolic reprogramming with increased glucose utilization and glycolysis, which was controlled by mitochondria to modulate cell proliferation and differentiation (39). When mitochondrial function is compromised during cellular stress, the bioenergy and responses of immune cells will be reduced. Indeed, a compromised mitochondrial function and deficient energy supply were observed in PBMCs from COVID-19 patients, which contributed to enhanced inflammatory responses causing disease severity (40). MD in T cells might also involve a series of metabolic dysregulation including reduced ATP-linked respiration, dampened glycolysis, and decreased mitochondrial membrane potential (35, 40). During these processes, the formation of oxysterols would increase mitochondrial oxidative stress (41), and in return, the exacerbation of mitochondria-derived ROS might lead to the rupture of redox homeostasis and the induction of apoptosis (42). To this end, we consistently found high calcium uptake and excessive production of mitochondrial ROS in T cells of COVID-19 patients. Under MD with excessive ROS and low mitochondrial membrane potential, fission is a critical way to remove damaged mitochondria *via* mitophagy (43). Whether or not SARS-CoV-2 infection dampens the activities of mitophagy and mitochondrial fission in host T cells as potential causes of MD remains to be further investigated. Furthermore, among patients with MD^hi^ T cells, our finding of poorer CD4 T_EM_ or memory CD8 T-cell responses to SARS-CoV-2 peptide or PMA stimulation highlighted the reduced antiviral T-cell immune responses. Our patients with MD^hi^ CD4 T_EM_ and memory CD8 T cells exhibited functional exhaustion with reduced production of pro-inflammatory cytokines such as TNFα and IFNγ, which was also in line with other reports on chronic hepatitis B or long-term tumor antigen exposure (44, 45). Interestingly, since CD4 and CD8 T cells might undergo different metabolic processes (46), it might explain why the frequency of MD^+^ T cells was correlated preferentially and respectively with CD4 T-cell loss and CD8 T-cell hyperactivation in this study. Further studies are required to reveal why more robust CD4 but not CD8 T-cell responses were found during the natural course of SARS-CoV-2 infection (21).

In terms of the association between MD and disease severity, it is possible that MD-related poor responses of CD4 T_EM_ and memory CD8 T cells might be linked with COVID-19 disease progression or severity. Profound T-cell loss during 6–10 days p.s.o. predicts ICU admission and disease severity (13, 15, 16), but it also potentiates thymopoiesis and results in naive T-cell overproduction in a compensative manner (47). Partially reversed T-cell count in severe patients during 11–15 days p.s.o. might be due to freshly compensated T cells. A recent single-cell transcriptomics study pointed out that SARS-CoV-2-specific CD8 T cells in mild patients were found to be pro-exhausted but those in severe APs were pro-survival (48), which might be a consequence of the compensation manner. Increased SARS-CoV-2-reactive T cells were also suggested as a cause of immunopathogenesis in patients (48, 49), which might be alleviated in patients with MD^hi^ T cells along with impaired T-cell functionality. Furthermore, most severe patients received treatment of steroid, when oxygen supply is required from the second week p.s.o (50). Unexpectedly, we found a relatively lower proportion of MD^+^ T cell but better T-cell functionality in CD4 T_EM_ and memory CD8 T cells of severe patients than mild ones during 11–15 days p.s.o. Steroid did not seem to be a distinct immunosuppressor for interrupting thymopoiesis and inhibiting T-cell responses (51), but early administration of steroid is probably good for CD8 T-cell restoration in CPs after day 14 p.s.o (52). It is, therefore, possible that MD in T cell may serve as a suppressive factor in immunopathogenesis during severity development.

## Data Availability Statement

The raw data supporting the conclusions of this article will be made available by the authors without undue reservation.

## Ethics Statement

The studies involving human participants were reviewed and approved by the institutional review board of University of Hong Kong/Hospital Authority Hong Kong West Cluster and Kowloon West Cluster Research Ethics Committee [UW 13-265 and KW/EX-20-038(144-26)]. The patients/participants provided their written informed consent to participate in this study.

## Author Contributions

ZC and K-YY supervised the collaborative teams and gained the research grants for this study. YM, RZ, and ZC designed the experiments, performed the experiments, collected the data, analyzed the data, interpreted the data, and wrote the article. KK-WT collected the clinical data, analyzed the clinical data, interpreted both clinical and experimental data, and prepared the article. LL, TC and CYHL participated in the experimental design, data analysis, and data interpretation. TC, HH, ZD, L-YY, and T-YL prepared samples from patients and healthy donors. KK-WT, JM-CC, TS-HC, DP-LL, OT-YT, ART and IF-NH collected the clinical samples and clinical data. YM, KK-WT, and RZ have contributed equally to this work.

## Funding

This work was supported by the Research Grants Council Collaborative Research Fund (grant number C7156-20GF, C1134-20GF, and C5110-20GF) in Hong Kong Special Administrative Region; Shenzhen Science and Technology Program (grant number JSGG20200225151410198); the University Development Fund and Li Ka Shing Faculty of Medicine Matching Fund from the University of Hong Kong to the AIDS Institute; the Innovation and Technology Fund, Innovation and Technology Commission, the Hong Kong Special Administrative Region Government; the National Program on Key Research Project of China (grant number 2020YFC0860600, 2020YFA0707500, and 2020YFA0707504); and generous donations including the Friends of Hope Education Fund. ZC’s team was also partly supported by the Theme-Based Research Scheme (grant number T11-706/18-N).

## Conflict of Interest

The authors declare that the research was conducted in the absence of any commercial or financial relationships that could be construed as a potential conflict of interest.

## Publisher’s Note

All claims expressed in this article are solely those of the authors and do not necessarily represent those of their affiliated organizations, or those of the publisher, the editors and the reviewers. Any product that may be evaluated in this article, or claim that may be made by its manufacturer, is not guaranteed or endorsed by the publisher.
