# Supplementary material for: Mitochondrial Dysfunction Associates With Acute T Lymphocytopenia and Impaired Functionality in COVID-19 Patients
Source: Front Immunol. 2022 Jan 14;12:799896. doi: 10.3389/fimmu.2021.799896 (PMC8795605; doi:10.3389/fimmu.2021.799896)
Supplement: Supplementary file 1 [file DataSheet_1.docx]

Supplementary Material

# Supplementary Figures.

**
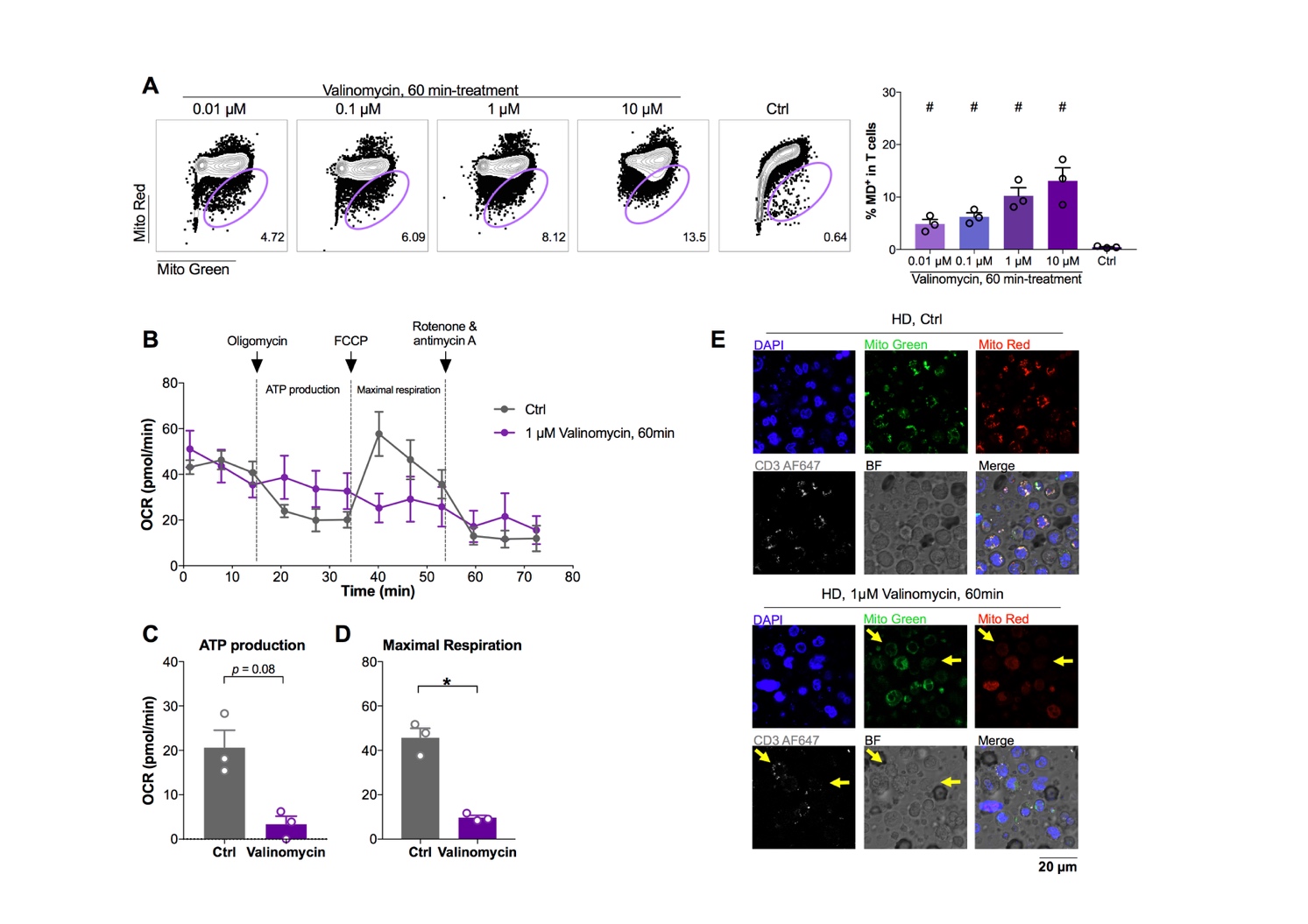
Supplementary Figure 1.** **MD profile in CD4 and CD8 T cells from healthy donors by different experimental methods.**

Freshly isolated PBMCs from 3 healthy donors (HDs) were treated with or without 0.01 µM, 0.1 µM, 1 µM and 10 µM Valinomycin for 60 min. (**A**) The percentage of MD^+^ T cells was detected using MitoTracker™ Green (Mito Green) and MitoTracker™ Red CMXRos (Mito Red) by flow cytometry. Data represents Mean ± SEM. Statistics were calculated based on One-way ANOVA test. # represents for *p*<0.05 when comparing with non-valinomycin control.

Purified total T cells from fresh PBMCs from 3 healthy donors (HDs) were treated with or without 1 µM Valinomycin for 60 min before performing Seahorse XF Cell Mito Stress Test. The oxygen consumption rate (OCR) along experimental time (**B**), ATP production (**C**) and maximal respiration (**D**) was analyzed and compared between valinomycin-treated group and non-treated control group. Data represents Mean ± SEM. Statistics were calculated based on paired Student’s t-test test. **p*<0.05.

PBMCs with 60-minute 1 µM valinomycin treatment were stained with MitoTracker™ Green (Mito Green, in green), MitoTracker™ Red CMXRos (Mito Red, in red) and anti-CD3 antibody (AF647, in white) for confocal study. Non-treated PBMCs serves as control. (**E**) Representative images from three independent donors were displayed. Yellow arrows points to MD^+^ T cells. Pixel size = 0.07 µM.


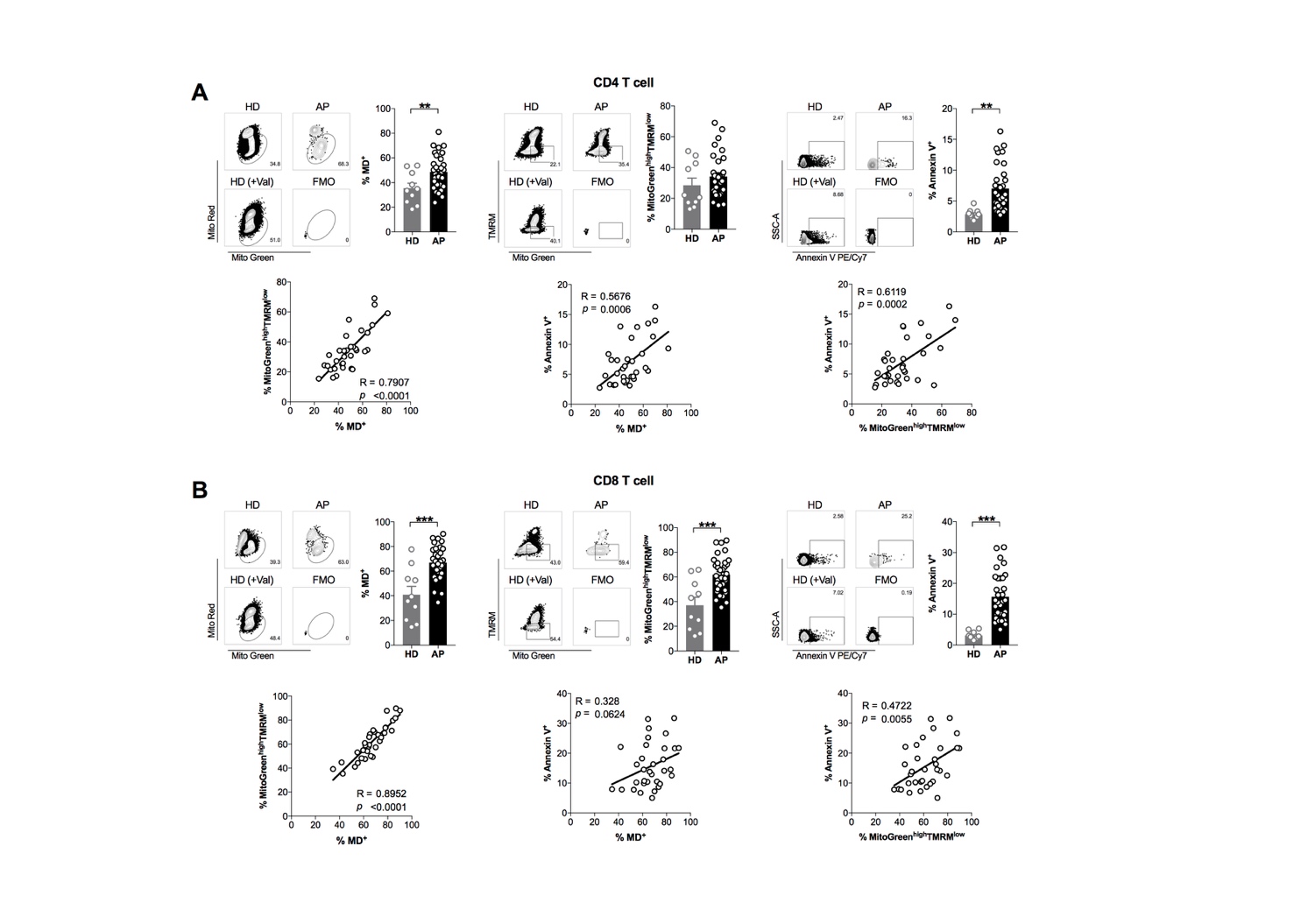


**Supplementary Figure 2. MD^+^ T cells is positively correlated with apoptotic proportion of T cells.**

Frozen PBMCs among 33 SARS-CoV-2 acute patients (APs) and 10 healthy donors (HDs) were thawed and used for further profiles on apoptosis and TMRM assay by flow cytometry. The percentage of MD^+^ cells, MitoGreen^high^ TMRM^low^ and Annexin V^+^ cells from both CD4 (**A**) and CD8 (**B**) T cells were compared between healthy donors and acute patients. Data represents Mean ± SEM. Statistics were calculated based on unpaired Student’s t-test test. **p*<0.05, ***p*<0.01, ****p*<0.001. (**A, B**) The correlation among these three indicators in acute patients was calculated by Pearson’s correlation coefficient analysis.


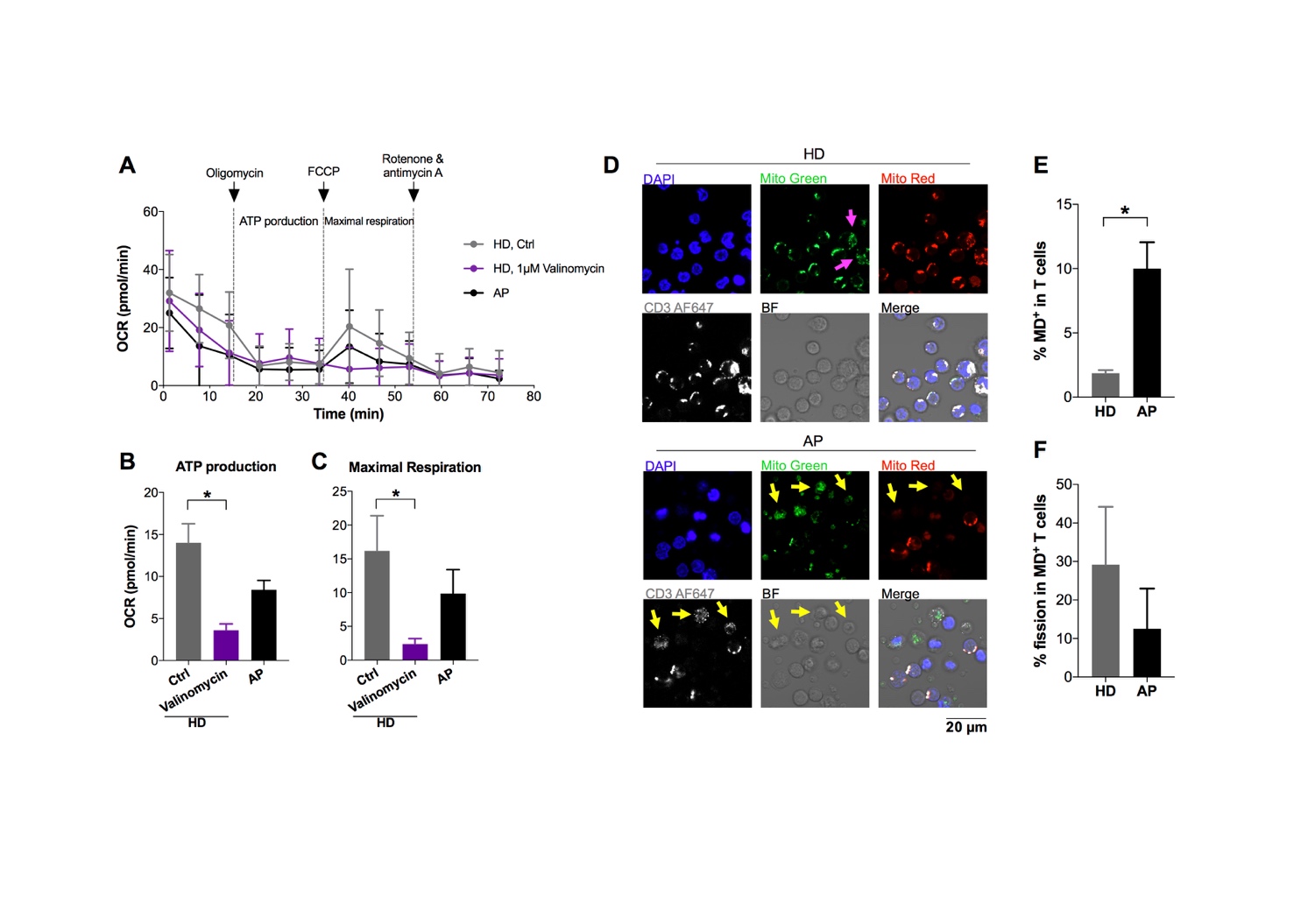


**Supplementary Figure 3.** **MD profile in CD4 and CD8 T cells from acute SARS-CoV-2-infected patients by different experimental methods.**

In Seahorse XF Cell Mito Stress Test, purified total T cells from 33 acute patients were pooled into 5 independent wells in order to obtain sufficient cells for the assay. Cells used in HD groups are also frozen cells and have pooled wells for control. Valinomycin-treated cells from HD were used for positive control, while non-treated cells from HD were used for negative control. The oxygen consumption rate (OCR) along experimental time (**A**), ATP production (**B**) and maximal respiration (**C**) were analyzed. Data represents Mean ± SEM. Statistics were calculated based on One-way ANOVA test. **p*<0.05.

PBMCs from HDs or APs were stained with MitoTracker™ Green (Mito Green, in green), MitoTracker™ Red (Mito Red, in red) and anti-CD3 antibody (AF647, in white) for confocal study. **(D)** Representative images from three independent donors were displayed. Yellow arrows points to MD^+^ T cells. Magenta arrows points to cells with fission morphology. Pixel size = 0.21 µM. The percentage of MD^+^ T cells (**E**) and the percentage of MD^+^ T cells with fission (**F**) in confocal study was calculated from at least 3 captured pictures from three independent donors.

**
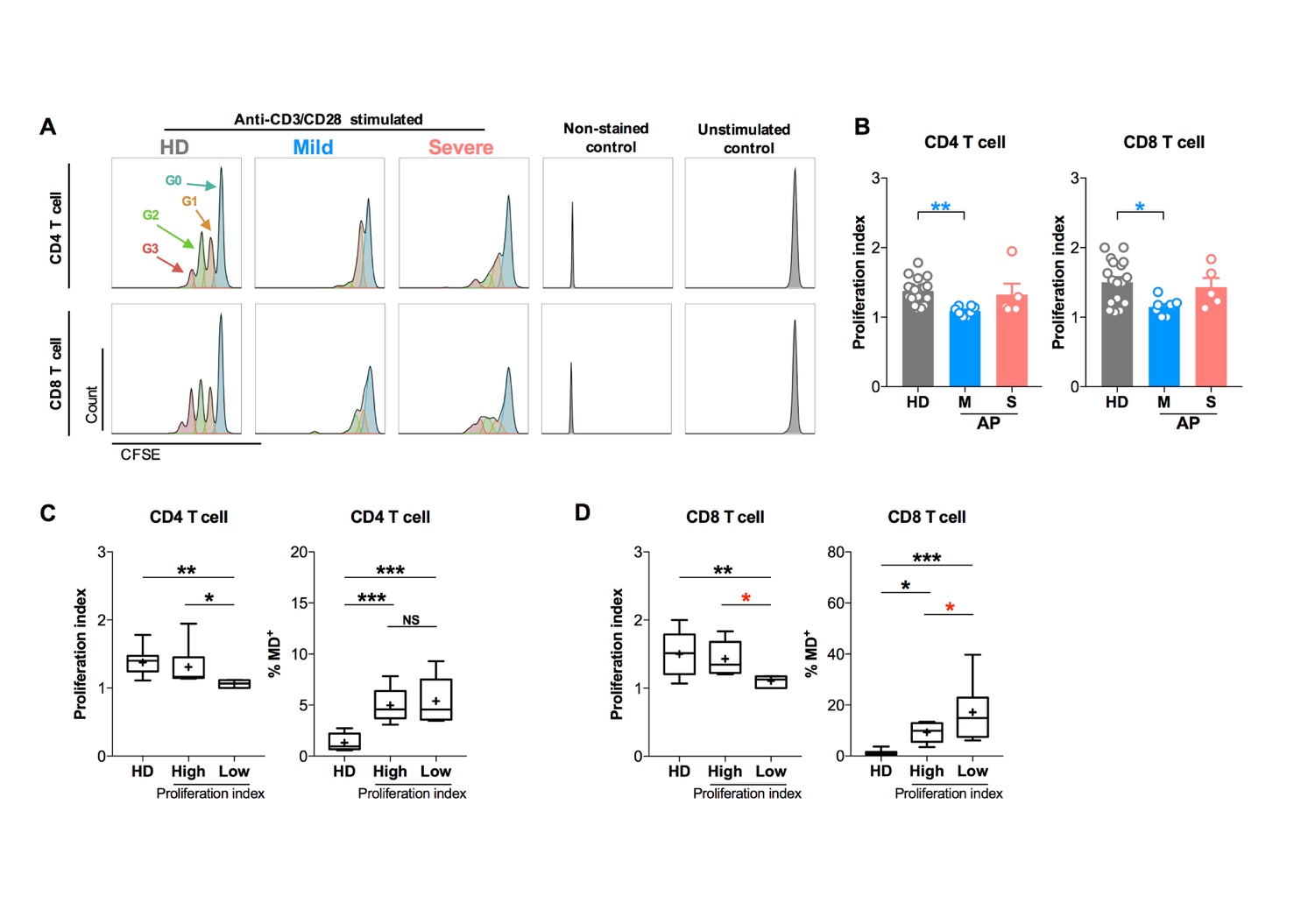
**

**Supplementary Figure 4. CD8 T cell with high MD proportion shows low proliferation potential upon pan activation.**

For proliferation assay, fresh PBMCs from 13 SARS-CoV-2 acute patients [APs; 8 mild (M) and 5 severe (S) ones on day 11-15 *p.s.o.*] and 18 healthy donors (HDs) were pre-stained with CFSE (5 µM) and then treated with anti-CD3 (2 µg/mL) plus anti-CD28 (1 µg/mL) antibodies for 3 days before FACS analysis in at least three independent experiments. (**A**) Representative histogram plots among groups were displayed. Cells that were not stained with CFSE served as non-stained control, while cells that were stained with CFSE but not stimulated served as unstimulated control. (**B**) The proliferation index of CD4 T cells and CD8 T cells from mild and severe APs was compared. HDs served as positive control. Proliferation index was calculated by normalizing total number of divisions [(G1/2)*1+(G2/4)*2+(G3/8)*3] to number of cells that went into division (G1/2 +G2/4 +G3/8). The proliferation index and the proportion of CD4 T cells (**C**) and CD8 T cells (**D**) with mitochondrial dysfunction (MD^+^) were compared between patients with high proliferation index (higher than median) and those with low one (not higher than median). HDs served as control. Data represents Mean ± SEM. Statistics were calculated based on One-way ANOVA test. **p*<0.05, ***p*<0.01, ****p*<0.001. NS = not significantly different.

**
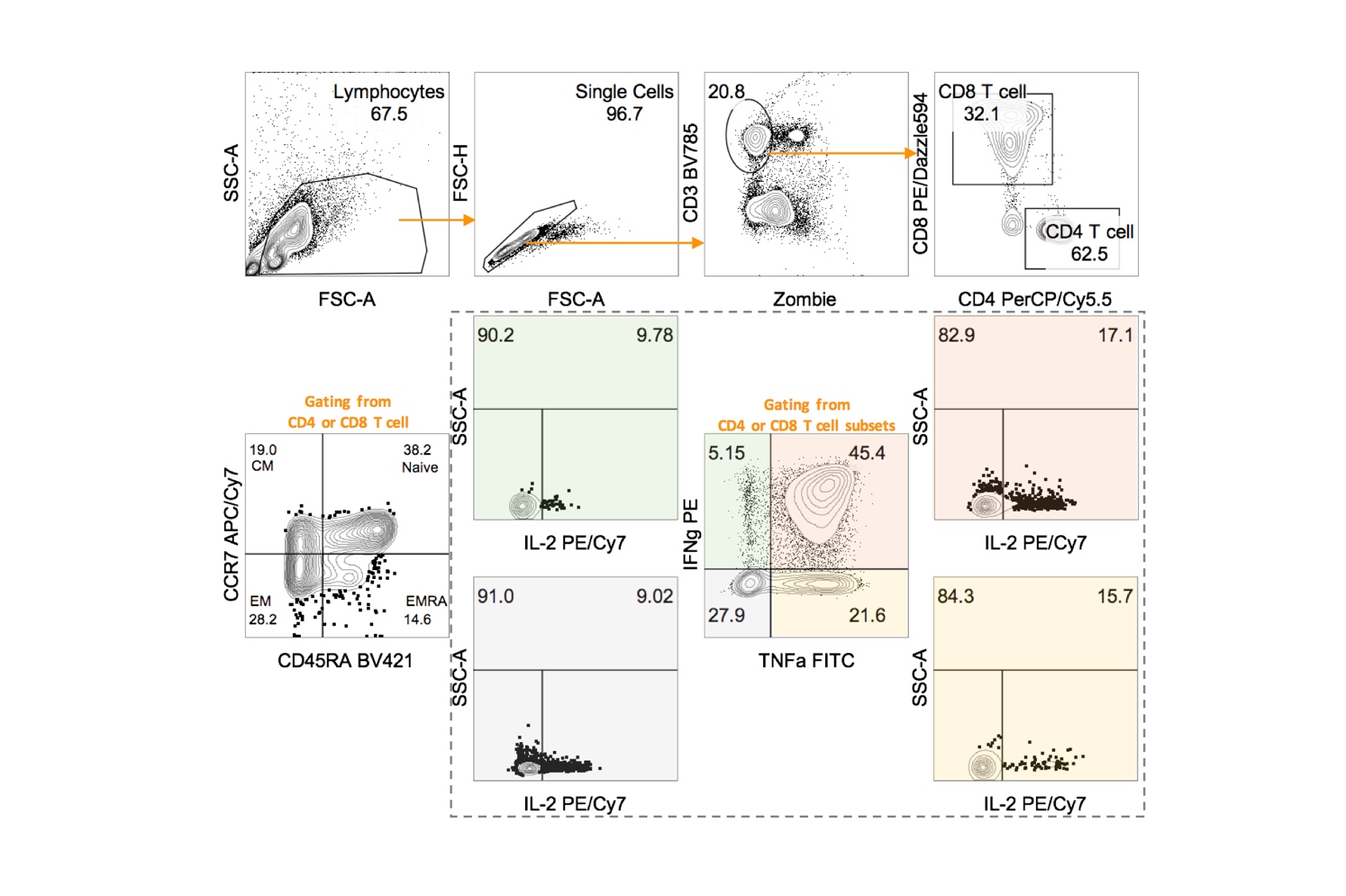
**

**Supplementary Figure 5. The gating strategy on TNF𝛂^+/-^IFN𝛄^+/-^IL2^+/-^ cells in CD4 or CD8 T cell subsets.**

13 SARS-CoV-2 acute patients [APs; 8 mild (M) and 5 severe (S) one on day 11-15 *p.s.o.*] and 18 healthy donors (HDs) were collected for polyfunctional analysis in at least three independent experiments. Fresh PBMCs from these 13 recruited patients were treated with PMA/Ionomycin in the presence of BFA for 6 hours. PBMCs from 18 HDs served as control. Cells were then harvested for FACS analysis on intracellular expression of TNFα, IFNγ, and IL-2. BFA-treated only cells served as negative control. Representative plots showed the gating strategy on TNFα^+/-^IFNγ^+/-^IL2^+/-^ cells in different subsets of CD4 or CD8 T cells (CM, Central memory cell; EM, Effector memory cell; EMRA, Effector memory cell re-expressing CD45RA).

**
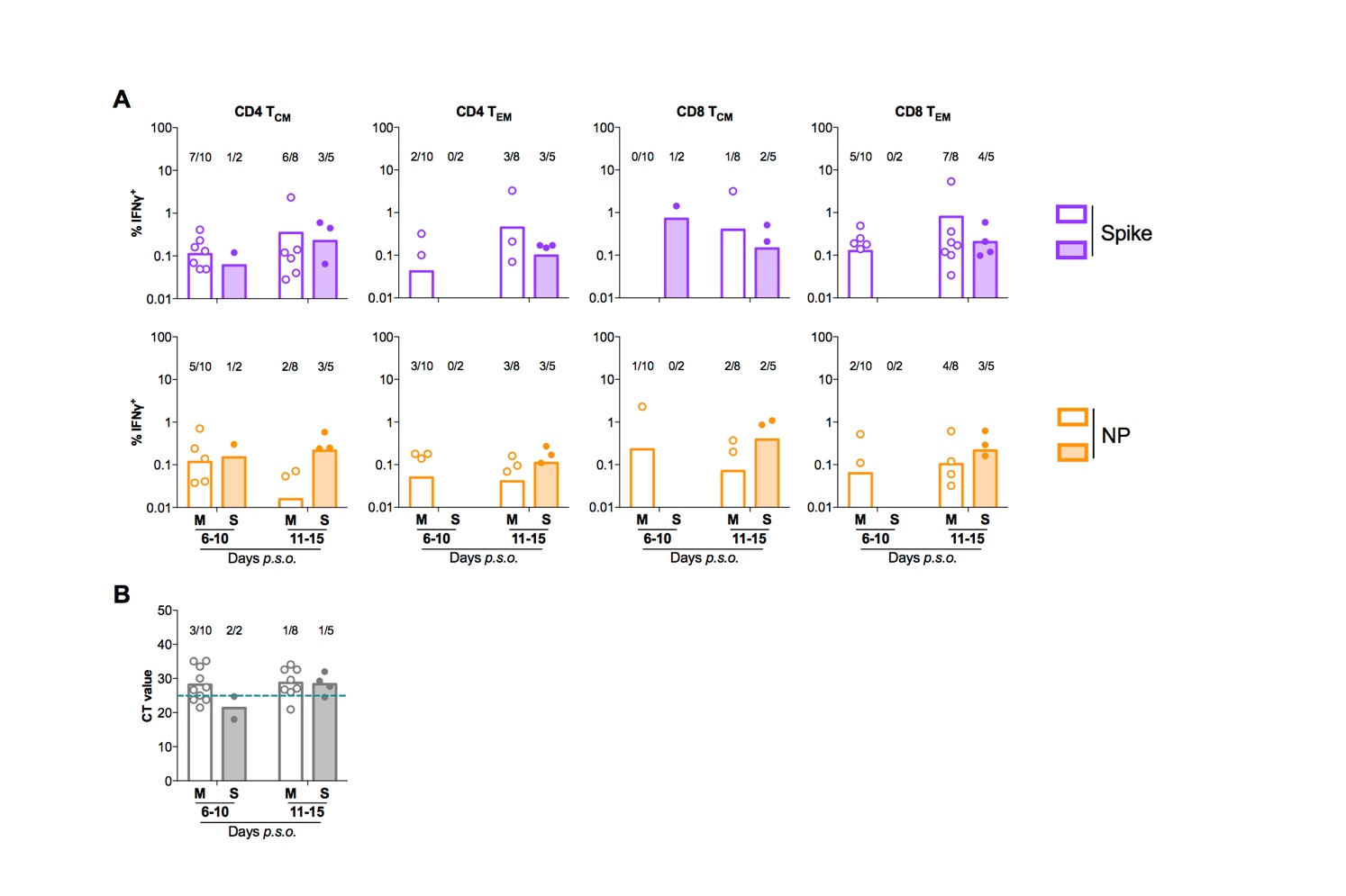
**

**Supplementary Figure 6. Antigen-specific T cell response in memory T cell subsets triggered by SARS-CoV-2 peptide pool.**

For detecting antigen-specific response, fresh PBMCs from 25 SARS-CoV-2 acute patients [APs; 18 mild (M) and 7 severe (S) ones during time period of 6-10 and 11-15 days *p.s.o.*] were treated with 1 µg/mL spike peptide pool or 5 µg/mL purified nucleocapsid peptide pool in the presence of 0.5 µg/mL anti-CD28 and anti-CD49d mAbs overnight. BFA was added at 6 h before cells were harvested for FACS analysis on intracellular IFNγ level. These 25 patients were separated into four groups based on days *p.s.o.* and disease severity. (**A**) The percentage of IFNγ^+^ cells in CD4 T_CM_, CD4 T_EM_, CD8 T_CM_ or CD8 T_EM_ cells in response to spike (in purple) or nucleocapsid protein (NP, in orange) peptide pool were displayed. Number above column represents number of responders in total patients in the certain group. (**B**) CT value from each patient was displayed. Number above column represents number of patients with CT value lower than 25 among total patients in the certain time point and group. Data represents Mean.

**
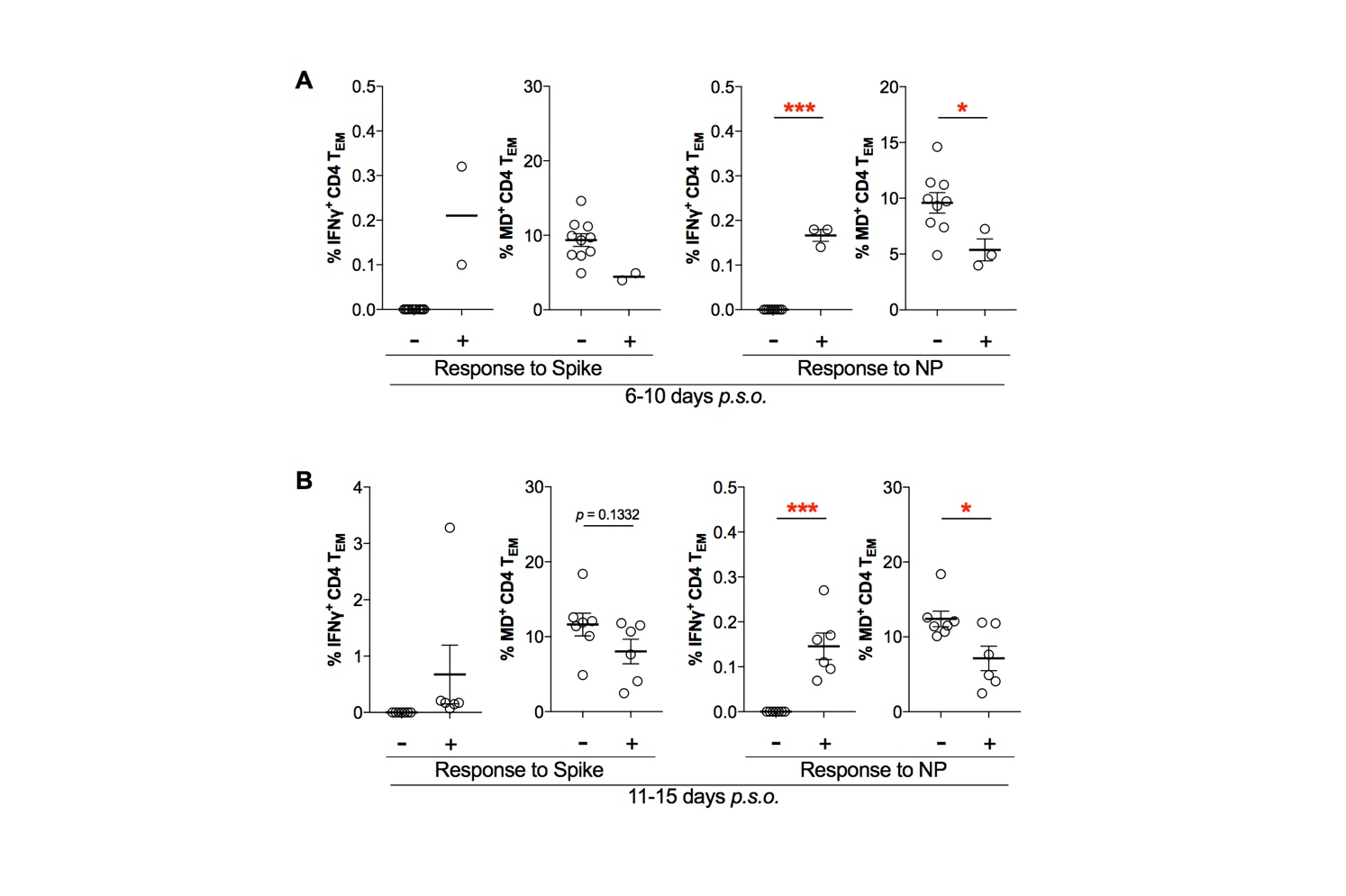
**

**Supplementary Figure 7. Patients with poor SARS-CoV-2-specific responses in effector memory CD4 T cells showed higher MD^+^ proportion.**

For detecting antigen-specific response, fresh PBMCs from 25 SARS-CoV-2 acute patients [APs; 18 mild (M) and 7 severe (S) ones during time period of 6-10 and 11-15 days *p.s.o.*] were treated with 1 µg/mL spike peptide pool or 5 µg/mL purified nucleocapsid peptide (NP) pool in the presence of 0.5 µg/mL anti-CD28 and anti-CD49d mAbs overnight. BFA was added at 6 h before cells were harvested for FACS analysis on intracellular IFNγ level. The percentage of IFNγ^+^ cells and MD^+^ cells in CD4 T_CM_, CD4 T_EM_, CD8 T_CM_ or CD8 T_EM_ cells from patients during the time periods of 6-10 days (**A**) or 11-15 days (**B**) *p.s.o.* was compared between poor responders (-) and good responders (+) specific to spike or NP peptide pool. Data represents Mean ± SEM. Statistics were calculated based on unpaired Student’s t-test. **p*<0.05, ***p*<0.01, ****p*<0.001.
